# Variable heteroplasmy of the Common 9-bp Deletion in the Human Mitochondrial Genome in Ancient and Present-Day Populations

**DOI:** 10.17912/micropub.biology.001482

**Published:** 2025-03-08

**Authors:** Flor C. Alcántar-Aguirre, Carlos Lozano-Flores, Maribel Hernández-Rosales, Alfredo Varela-Echavarría

**Affiliations:** 1 Instituto de Neurobiología, Universidad Nacional Autónoma de México (UNAM); 2 Centro de Investigación y de Estudios Avanzados (CINVESTAV) Unidad Irapuato

## Abstract

The recurrent 9-bp deletion in the intergenic region between COII and tRNA _Lys_ genes of the human mitochondrial genome is present in various world populations and has been linked to disease. The heteroplasmy of this deletion in the different carrier mitochondrial lineages, however, has remained largely unexplored. Employing deep sequencing mitochondrial DNA data, we quantified the deletion in diverse ancient and present-day human populations. We observed low 9-bp deletion heteroplasmy in specific haplogroups of ancient populations from various continents, high levels in their closely-related present-day populations, and independent emergence at high levels in isolated present-day lineages, always without reaching complete homoplasmy.

**Figure 1. Variable heteroplasmy of deletions in the human mitochondrial genome and phylogeny of present-day and ancient carrier populations of the 9-bp deletion:**
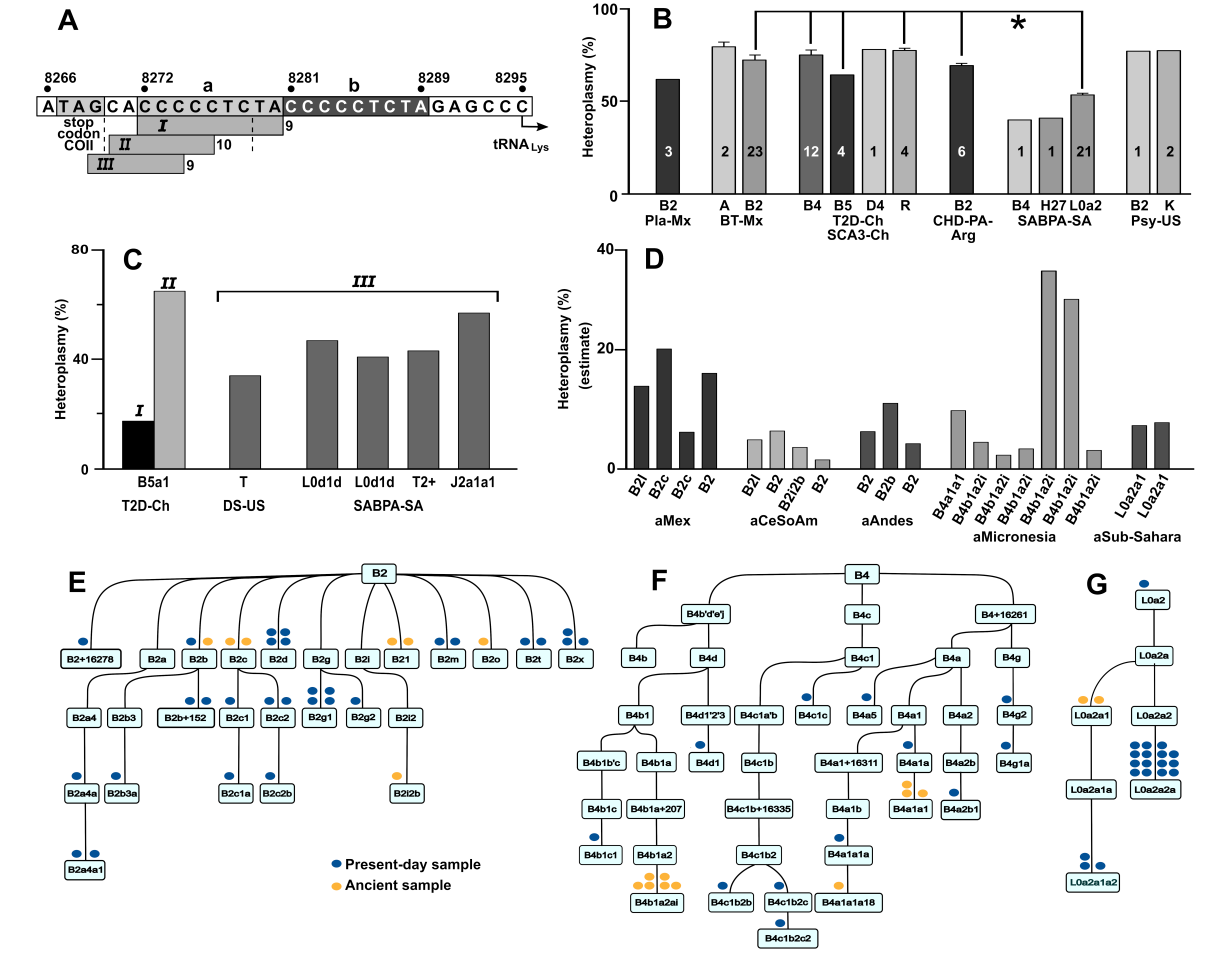
**(A)** Deletions were detected in the intergenic region between the cytochrome oxidase II (COII) and the tyrosine tRNA (tRNA _Lys_ ) genes. The mitochondrial genome nucleotide sequence is depicted in the long horizontal box with genomic coordinates. The termination site for COII is at position 8267, while tRNA _Lys_ initiates at 8295. Dual direct repeat motifs within this area are highlighted by shaded boxes "a" and "b". Below the nucleotide sequence, three horizontal bars illustrate the deletions identified within this region. Bar *I* denote the 9-base pair (9-bp) deletion (coordinates 8271-8280). Bars *II* and *III* correspond to two additional deletions found at high heteroplasmy (8271-8281 and 8269-8278, respectively). The length of the deletion is indicated to the right of each bar. ( **B)** Heteroplasmy of the 9-bp deletion in different haplogroups in present-day human populations. Average heteroplasmy is indicated for all individuals with high heteroplasmy of the different haplogroups for each of the datasets indicated at the bottom of the figure (Pla-Mx, BT-Mx, T2D-Ch/SCA3-Ch, CHD-PA-Arg, SABPA- SA, and Psy-US). Each bar represents a haplogroup and indicates the number of individuals exhibiting the 9-bp deletion. Above each bar, the standard error (SEM) is indicated for haplogroups with at least 4 individuals. The asterisk (*), indicates a significant difference between the heteroplasmy level of L0a2 samples (SABPA-SA) and B2 (BT-Mx and CHD-PA-Arg), B4, B5, and R (T2D-Ch/SCA3-Ch) samples (p<0.05). ( **C** ) Additional deletions were detected in the COII-tRNA _Lys_ intergenic region in present-day human populations. Deletions in this graph correspond to those shown in panel A. In the T2D-Ch dataset the 9-bp (deletion *I* ) was found together with the deletion *II* in the same individual. In the DS-US and the SABPA-SA datasets, deletion *III* was found in the absence of deletions *I* or *II* . Para-haplogroups or haplogroups are indicated for each individual below each bar. ( **D** ) Estimate of heteroplasmy of the 9-bp deletion in ancient populations from diverse world regions. Dataset identifiers for the different studies are indicated below each group of samples (aMex, aCeSoAm, aAndes, aMicronesia, and aSub-Sahara). Each bar represents an individual sample, and its haplogroup is indicated below. **(E-G)** Phylogenies of haplogroups of present-day or ancient samples carrying the 9-bp deletion. The phylogenies are shown for haplogroups B2 (E), B4 (F), and L0a2 (G). Only branches containing samples with the deletion are displayed for each haplogroup. Blue dots above the box of individual haplogroups represent present-day samples, while yellow dots indicate ancient samples.

## Description

The human mitochondrial genome is prone to inherited or spontaneous mutations, including point mutations, deletions, and insertions, which contribute to various severe, often lethal diseases with a prevalence greater than 1 in 3500 individuals (Gorman et al., 2015). A common deletion in the mitochondrial genome is the loss of the 9-base pair (9-bp) sequence CCCCCTCTA located between the cytochrome oxidase II (COII) gene and the tRNA _Lys_ gene (Deletion I, Fig. 1A ). Initially identified in East Asian populations (Wrischnik et al., 1987), this deletion has since been observed in other world populations (Watkins et al., 1999; Yao et al., 2000). Although it has been associated with diseases such as mitochondrial disorders, hearing loss, and polycystic ovary syndrome (Borgione et al., 2013; Jin et al., 2012; Zhuo et al., 2010), the level of heteroplasmy and frequency of this deletion across different populations remain underexplored. This work addressed these issues by employing a quantitative approach to analyze mitochondrial DNA datasets obtained by massive parallel sequencing from present-day and ancient human populations from geographic locations where the deletion has been previously detected.

In present-day populations, we analyzed mitochondrial DNA from datasets that contain samples of healthy individuals and individuals with several diseases from the Americas, Asia, Africa, and Europe. We observed the 9-bp deletion in seven of the nine datasets with elevated frequency and heteroplasmy ranging from 40% to 80% in individuals of specific haplogroups. Figure 1B represents all the samples carrying the deletion with at least 15% heteroplasmy from all datasets. In the Pla-Mx dataset, composed of 8 samples of mitochondrial Amerindian lineages A-D from Mexico, only the three B2 samples revealed the deletion with a homogeneous heteroplasmy of 62%. Our findings were supported by capillary sequencing of PCR-amplified fragments from each of the three samples, which confirmed the recurrent 9-bp deletion. Of the BT-Mx dataset, also from Mexico, every sample within the B2 sub-haplogroups (23 samples) exhibited the deletion with an average heteroplasmy of 74%. Moreover, out of 42 samples not ascribed to the B2 lineage, the deletion was present in only two A samples at an average of 80%. In the Chinese collections T2D-Ch and SCA3-Ch, high heteroplasmy was detected in all individuals of haplogroups B4 (12 in total) and B5 (4 in total), at 75% and 65% heteroplasmy, respectively. A lone D and four R samples had the deletion at 78% heteroplasmy from the 89 non-B samples assessed. The Argentinian CHD-PA-Arg dataset comprised 17 mother-offspring sample pairs predominantly ascribed to A2, B2, and D1 Amerindian haplogroups and some of Euro-Indian H, T, and U lineages. Of this dataset, only the three B2 pairs had the deletion with 69% heteroplasmy. In the South African SABPA-SA dataset, 200 samples were identified as belonging to the African L para-haplogroup, while 162 were of European and Asian lineages. All samples of L0a2 sub-haplogroups (21 in total) bore the 9-bp deletion, while only single B4 and H27 samples carried it, at heteroplasmy of 53%, 40%, and 41%, respectively. Within the Psy-US dataset composed of samples from 41 individuals predominantly of Amerindian, European, and African haplogroups, the two individuals from the K para-haplogroup and a sole B2 individual displayed the deletion, with heteroplasmy values of 78% and 77%, respectively. In contrast, the DS-US and UC-US datasets, notably excluding B and L samples, did not have the deletion at high heteroplasmy. Statistical analysis revealed no significant heteroplasmy differences between B2 haplogroups from different datasets (Pla-Mx, BT-Mx, CHD-PA-Arg, and T2D-Ch) and R haplogroups ( Figure 1B ). The heteroplasmy of SABPA-SA L0a2 samples, however, was significantly lower.

It is important to note that the cohorts under study were not selected for disease correlation. The SABPA and Pla-Mx datasets were constructed with no link to any particular disease, others had small numbers of disease samples, or their subcategories had even fewer members. Consistent with this, no increase in disease frequency was linked to the deletion (Wilcoxon test, p < 0.05).

Remarkably, in the T2D-Ch, DS-US, and SABPA-SA datasets, two additional high-heteroplasmy deletions were identified in the same intergenic region. Deletion II (8271-8281)( Fig. 1A ) was found in a healthy B5a1 haplogroup individual with 65% heteroplasmy, alongside the 9-bp deletion at 15% heteroplasmy ( Figure 1C ). Deletion III (8269-8278)( Fig. 1A ) was observed in a T haplogroup Down Syndrome patient with 34% heteroplasmy and four L, T, and J SABPA-SA samples with heteroplasmy ranging from 41% to 57%, all in the absence of deletion I ( Figure 1C ).

Analysis of the 9-bp deletion in ancient mitochondrial DNA allowed us to make only an estimate of the heteroplasmy due to the low depth of coverage with this type of sample ( Fig. 1D ). The deletion was present in the B2 samples from Mexico (aMex), with estimated heteroplasmy ranging from 6% to 19%, and in B2 samples from Brazil, Chile, and Peru at 1.5%-11.6% (aCeSoAm and aAndes). Meanwhile, the deletion was observed in B4 samples from Micronesia at 2% to 37% heteroplasmy (aMicronesia), and in L0a2a1 sub-haplogroup samples from Sub-Saharan Africa, it peaked at 7.9% (aSub-Sahara). The 9-bp deletion was absent in samples from East Asia.

Phylogeny analysis revealed that the B2 samples bearing the deletion from present-day and ancient datasets were widely distributed in diverse sub-lineages in this haplogroup ( Fig. 1E ). B4 samples in present-day and ancient carriers also had a widespread distribution despite their small number ( Figure 1F ), while in the L0 haplogroup, all present-day and some ancient carriers were found restricted to the L0a2 lineage ( Figure 1G ).

Our results provide precise measures of the heteroplasmy of the 9-bp deletion in different genomic mitochondrial lineages of diverse world regions. Previous studies have used the presence of the 9-bp deletion to define the B2 haplogroup among Aztecs (Mata-Míguez et al., 2012), and the B para-haplogroup in Amerindian lineages (Alves-Silva et al., 1999; Demarchi et al., 2001; González-Oliver et al., 2017; González-Sobrino et al., 2016; Smith et al., 1999; Wester et al., 2020). Consistent with these reports, we find that all present-day B2 samples analyzed carry the deletion, with the novel finding of a remarkably high heteroplasmy which appears to have increased from earlier Amerindian B2 ancestors. A higher heteroplasmy was also observed in all present-day B4 individuals from China compared to ancient B4 samples from diverse Asian regions. Based on their phylogeny, it can be inferred that a B4 Asian sub-haplogroup carrying the 9-bp deletion was likely the ancestor of the closely related B2 Amerindian founders. The absence of the deletion in present-day B5 individuals (Thangaraj et al., 2005), however, suggests that the B para-haplogroup is unlikely to have carried the deletion homogeneously. Among L para-haplogroup samples, the deletion was found at high heteroplasmy only in all present-day L0a2 samples from South Africa analyzed and at low heteroplasmy or absent in other L0, L1, and L3 individuals. Since the deletion was also found in two prehistoric L0a2a1 Sub-Saharan samples at low heteroplasmy, it is plausible that the deletion arose in L0a2 ancestors of present-day populations. On the other hand, the finding of the deletion at high heteroplasmy in A2 individuals in present-day datasets from Mexico and Argentina and its absence in cognate A2 samples support the idea that the deletion also arose independently in the American continent (Torroni et al., 1994). A similar independent origin is hinted at by the presence of the deletion in a D4b1b individual and its absence in other D4 individuals from China, as well as its presence in European ancestry individuals of H, R, and K lineages.

Hence, the results of this work reveal a robust transmission of the deletion at high heteroplasmy in B2, B4, and L0a2a haplogroups despite the strong genetic drift the mitochondrial genome is constantly exposed to. This exploratory investigation reveals a complex evolutionary dynamics that suggests the 9-bp deletion increased in abundance to the high heteroplasmy observed in these lineages. In no instance, however, the deletion was found at homoplasmy in any dataset, which is possibly the result of functional selection of a minimal fraction of wild-type mitochondrial genomes in carrier individuals.

We recognize that the inherent technical difficulties associated with ancient DNA samples preclude a reliable one-to-one comparison with present-day samples. Additional studies are thus needed to enrich the quantitative view of the presence and dynamics of the 9-bp deletion.

An additional consideration is that the use of datasets containing disease samples may have skewed the results in some cases, although no correlation was detected between the 9-bp deletion and disease at the population or haplogroup level. Nevertheless, future studies intended to assess the link of this deletion with disease must take into account genomic mitochondrial lineage and heteroplasmy with the solid grounding of our findings.

## Methods

Datasets

Owing to the limited availability of deep sequenced samples of mitochondrial DNA, especially from different regions of the Americas, datasets that were used included some from studies on diverse diseases with no apparent or suspected link to the deletion. Hence, some datasets contain samples of healthy individuals and some of diseased individuals as well.

The placenta set (Pla-Mx) contains eight samples of healthy donors of self-declared Mexican ethnicity (NCBI SRA Bioproject PRJNA1008659 ) (Varela-Echavarría et al., 2024). The BT-Mx dataset consists of 92 paired breast tumor and blood samples of Mexican women (PRJEB40354) (Pérez-Amado et al., 2020). Samples within the SCA3-Ch collection, procured from mainland China, contain 31 healthy controls and 38 patients with spinocerebellar ataxia ( PRJNA590078 ) (Yuan et al., 2020). The T2D-Ch dataset, from a study in the Shenzhen and Hunan regions, contains 30 healthy controls and 10 patients with type 2 diabetes ( PRJNA563929 ) (Li et al., 2022). The CHD-PA-Arg dataset is from a study conducted in Argentina comprising 17 sample pairs of mother and offspring which had either congenital heart malformations or palatal irregularities except for one pair which had neither ( PRJNA636010 ) (Rebolledo-Jaramillo et al., 2021). The DS-US dataset, rooted in a study centered around Buffalo, NY, was collated from diverse US repositories and contains samples of 33 control donors and 12 donors with Down Syndrome ( PRJNA319813 ) (Hefti et al., 2017). The SABPA-SA dataset, from the cohort Sympathetic Activity and Ambulatory Blood Pressure in Africans, was put together in South Africa, and comprises 362 individuals of both African and European lineages recruited prospectively and not based on any disease ( PRJNA403942 ) (Malan et al., 2015). The UC-US dataset, based on samples sourced from Seattle, Washington contains colon biopsies from 3 non-ulcerative colitis control patients, 4 with ulcerative colitis with cancer, and 3 with ulcerative colitis without cancer ( PRJNA449763 ) (Baker et al., 2019). The Psy-US dataset comprises paired tissue specimens from 41 individuals, gathered from El Paso, TX, and Irvine, CA with various psychiatric diseases, except two controls ( PRJNA486215 ) (Hjelm et al., 2019).

The following ancient samples were also analyzed: The aMex dataset comprises samples of central Mexico dated between 670-1350 years of the current era (PRJEB51440) (Villa-Islas et al., 2023). The aCeSoAm dataset is composed of samples from Brazil, Chile, and Peru, dated 1200-600 years before the present (BP) (PRJEB28961) (Posth et al., 2018). The aAndes dataset contains samples from Peru dated 600 BP (PRJEB37446) (Nakatsuka, Lazaridis, et al., 2020). The aPatagonia dataset contains samples from South Patagonia from approximately 5800 to 100 BP (PRJEB39010) (Nakatsuka, Luisi, et al., 2020). The aMicronesia dataset contains samples from Micronesia dated 2800 to 200 BP (PRJEB51180) (Liu et al., 2022). The aEast Asia dataset contains samples from diverse regions in East Asia dated between 8000-1000 BP (PRJEB42781) (Wang et al., 2021). The aSub-Sahara dataset contains samples from Sub-Saharan Africa spanning approximately the past 18,000 years (PRJEB32086, PRJEB49291) (Lipson et al., 2020, 2022) and from prehistoric Africa (PRJEB21878) (Skoglund et al., 2017). These samples were treated with uracil-DNA-glycosylase (UDG) to reduce the rate of characteristic ancient DNA damage, were prepared using mitochondrial genome capture to reduce genomic and environmental DNA, and were screened for modern mitochondrial genome contamination which ranged from 2% to undetectable and was expected to have a negligible impact on the results of this study.

Cloning and sequencing of the 9-bp deletion in placental samples

The COII-tRNA _Lys_ intergenic region was amplified by PCR with the primers IDC-2a (5'-CGGGGGTATACTACG) and IDC-2b (5'-CCATACGGTAGTATTTAGTTG) for placental samples with the 9-bp deletion. This yielded a 242 base-pair fragment from each sample which was ligated into the pGEM-T Easy vector (Promega, Madison, WI). Three clones were selected from each sample and were sequenced with the Applied Biosystems AB3730 DNA Analyzer at LANGEBIO in Irapuato, Mexico.

Sequence data analysis

Raw sequencing data from all the datasets underwent initial quality assessment using FastQC (www.bioinformatics.babraham.ac.uk/projects/fastqc). Sequences of insufficient quality were discarded. The remaining sequences were subsequently refined using Trimmomatic (Bolger et al., 2014), to trim off suboptimal ends. To map the datasets, Segemehl (Otto et al., 2014) was employed, using the revised Cambridge Reference Sequence (rCRS) of the human mitochondrial genome (Andrews et al., 1999; Danecek et al., 2021), making use of the split-read option. Variants were detected using mutect2 (https://gatk.broadinstitute.org/hc/en-us/articles/360037593851-Mutect2), setting the following specific parameters: minimum base quality score of 28, median mapping quality of 50, global read mismapping rate of 28, excluding soft-clipped reads. Other parameters were fine-tuned to eschew limitations related to sequencing depth or variant frequency, while the remainder were left at their default settings. Modern sample haplotypes were ascertained using Haplogrep 3 (Schönherr et al., 2023) with phylotree-rcrs@17.2, which employed the vcf files from mutect2. Corresponding geographical or ethnic designations of the resulting haplogroups were then traced on PhyloTree (van Oven & Kayser, 2009) and the relevant tree fragments were extracted from the Haplogrep 3 clusters. Ancient DNA sample haplotypes were referenced from their original publication sources. Due to the circular DNA extraction method that minimized nuclear DNA presence, NUMT contamination in placental samples was previously determined to be inconsequential (Varela-Echavarría et al., 2024). Furthermore, the mitochondrial DNA-specific enrichment reduced the potential NUMT contamination in the other datasets.

To discern deletions in each present-day sample, the haarz tool within Segemehl was used with default values to identify those that had a minimum of 5 supporting reads and a split-read mapping score threshold of 25. This allowed the identification of all possible deletions throughout the mitochondrial genome including low frequency events. Since we concentrated our analysis in the high heteroplasmy carriers, in a later step a more stringent filter was applied to select only those with values of at least 15% (see below). After this step, the number of reads supporting the deletions in each of the selected samples ranged from 102 to 23665, which strengthens our conclusion that the deletion calls are accurate.

Owing to their inherently lower sequence quality, deletions in ancient samples were evaluated using a reduced split-read mapping score of 20. Moreover, to test whether the detected deletions included false-positive events, we also assessed deletions with a split-read score of 15. This resulted in the discovery of additional deletion-carrying reads only in B2 and B4 samples of the CeSoAm and Micronesia datasets, respectively, which had revealed deletions with the more stringent score of 20. Hence, the deletion calls in ancient samples are likely reliable.

Coverage depth across the mitochondrial genome was gauged for all samples using samtools (Danecek et al., 2021) utilizing the sorted bam output from Segemehl with the -s option for paired-end read samples. This option was not applied for Psy-US, SABPA-SA, and ancient samples due to the use of single-end reads. The average depth was then evaluated both for the entire mitochondrial genome and for the 50-nucleotide regions flanking the two direct repeats at the intergenic region. This local depth was employed as a benchmark to estimate the heteroplasmy of the identified deletions. Local depth of the samples carrying the deletion in each present-day dataset averaged from 1127 to 83369 and for ancient samples from 22 to 149.

Statistical analysis

To determine whether there was any association of the presence of the 9-bp deletion or heteroplasmy level to the different diseases in the case-control datasets, the Wilcoxon signed rank test was employed (two-tailed, p ≤ 0.05). Additionally, the heteroplasmy levels of the different haplogroups with 4 samples or more ( Figure 1B ), were analyzed in a completely randomized design using the general linear model procedure (SAS version 9.3 software; SAS Institute, Cary, NC, USA) with the significance level set at p ≤ 0.05. The method of least square means ± SEM was used to analyze differences between treatments. Data are presented as mean ± standard error of the mean (SEM).
